# Integrated Care in Action: A Practical Guide for Health, Social Care and Housing Support: Robin Miller, Hilary Brown and Catherine Mangan, London: Jessica Kingsley Publishers, 2016, pp 232, ISBN: 978-1-84905-646-5 (paperback)

**DOI:** 10.5334/ijic.4199

**Published:** 2018-08-06

**Authors:** Henriikka E. Laurola

**Affiliations:** 1 International Foundation for Integrated Care, UK

**Keywords:** integrated care, health care, social care, housing support, building blocks, person-centered care, care management, leadership

This book, authored by Dr Robin Miller, Hilary Brown and Catherine Mangan of the University of Birmingham, sets out to examine the current academic and practical knowledge of integrated care in support of those who lead integration in health, social care and housing support. It specifically delves into the ‘why’, ‘what’ and ‘how’ of integrated care and explores how common challenges in all levels of service provision may be overcome. The book makes a clear contribution to integrated care by providing an all-in-one toolkit combining evidence, theory and practice pertaining to its design and implementation. In 10 chapters *Integrated Care in Action* covers the whole process of designing, developing, sustaining, and evaluating integrated care. Additionally, the book provides definitions of key terms, an appendix of further resources, as well as subject and author indexes. Each chapter contains case studies to demonstrate the book’s teachings in practice. The tools, theories and techniques presented are also generally accompanied with helpful illustrations. The following five paragraphs of this review will provide an overview of the book’s chapters. The two final paragraphs assess the book as a whole, by discussing its strengths, weaknesses, and target audience.

The book begins with a short preface explaining the publication’s aims, approach and contents. To ease the step-by-step path of leading an integrated care initiative, the authors have organised the book chapters according to their understanding of the key building blocks of integrated care, presented in the preface. While it is noted that integration should not be considered as a linear process, but rather as an overlapping and interactive one, the building blocks recur in the titles of the Chapters 2–9. The introduction provides a concise explanation why integrated care is important, the various ways of understanding it, as well as the aims, benefits and evidence related to integrated care. Additionally, the introductory chapter addresses the common uncertainties and frustrations related to integration by suggesting what has gone wrong in earlier attempts to integrate care. The reassuring tone present throughout the book guides the reader to turn barriers into enablers and doubts into confidence.

Chapter 2 introduces the first building block of integrated care – ‘Establishing Purpose and Need’ – and encourages the reader to set the groundwork for an effective, tailored approach by providing a set of perspectives, organisational development tools and methodologies for examining and mapping the ‘wicked issues’ for which integrated care is sought as a solution. The authors inspire to imagine the desired outcomes and be creative in designing an integrated care initiative. Chapter 3, ‘Engaging and Involving Individuals and Communities’, explains and emphasises the importance of involving service users, carers and citizens into activities in micro, meso and macro levels of integrated care. Again, barriers and enablers to engagement are discussed and key stages of involvement are explored through case examples.

Chapter 4, ‘Leading Self and Others’, delves into forms of leadership and sources of power in leading integrated care. The reader is encouraged to recognise the complexity of his/her environment and to find ways to tackle wicked issues whilst accepting conflict and ambiguity. The common perspective of leading others has here been extended into leading the self; the chapter reminds the reader of the importance of recognising stress and surviving change. Various frameworks to help in leading change in a complex environment are introduced, such as transactional vs. transformational leadership and systems leadership, accompanied by practical examples. Chapter 5, ‘Managing Change – Processes and People’, cuts into the means by which the change processes can be managed in a desired and more predictable way in an organisation. In addition to presenting useful project management approaches, the chapter highlights the significant influence of key stakeholders into the change processes. The soft aspects the authors recommend include nurturing organisational learning, trust and good communication as well as understanding and managing the inevitable change-related anxiety of staff.

Chapter 6, ‘Evaluating and Reviewing Integration’, encourages leaders of integrated care to recognise the purpose and focus of a review undertaken before approaching the task. The key practical issues to be sorted before getting started with evaluation are listed and the ethics shortly discussed. The introduced common methods and tools to lead research and analyse data are again supported by case examples. Moreover, Chapters 7 and 8, ‘Working with Service Users and Carers’ and ‘Working with Staff’, discuss the ways to truly engage service users and staff in service design by enabling them to contribute their experience, knowledge and skills. Regarding service users and carers, the asset-based viewpoint of the authors highlights peer support, self-care and -management, and in terms of staff, interventions positively affecting and taking into consideration organisational culture and the needs of (inter-professional) team work.

Chapter 9, ‘Working with Processes and Systems’, moves from the individual and team levels into the supporting aspects. While it is noted that no one system or process approach will achieve integration, various ones can support it depending on the organisation’s needs, integration aims and desired outcomes. Examples such as IT, pooled budgets, care coordination and record keeping are presented and reviewed. The final building block ‘Sustaining and Improving’ in Chapter 10 describes how it can be ensured that an integrated care initiative keeps its focus and continues to improve. Aspects of leadership, engagement and resources are again highlighted. The reader learns that the end of an initiative does not need to be or should be the end of key transitions for different stakeholders.

All in all, *Integrated Care in Action* is well-written, easy to read and – with the practical case examples and the sparing use of academic references – easily approachable to a wide audience in practice-oriented roles. The book’s strength is in the tools, techniques and methodologies with which it provides the reader; these persist through time while academic and practical knowledge of integrated care develops and grows. The way the authors address the frustrations and anxieties related to integration is important since it keeps a doubtful reader interested and supports the reader despite possible earlier set-backs. Unlike many other publications in the field, the book identifies the significant role of community-based organisations as producers of integrated care in addition to the public health and social care sector. Furthermore, the importance of co-production and service user engagement is highlighted throughout the book. The structure arranged according to the building blocks of integrated care is clear and allows the reader to effortlessly find their topic of interest. The appendix of additional materials, as well as the helpful illustrations, complement the well-defined building blocks. A particular merit of the books is that it learns from both successful and unsuccessful integrated care initiatives and places an emphasis on the complexity of the operating environments. This represents the current realities in the field and guides the reader to overcome persistent barriers to develop and maintain integrated quality care.

While the authors target the book primarily towards leaders in the domain of integrated care, the book provides a comprehensive yet easy-to-grasp introduction to integrated care for students, care practitioners or indeed anyone interested in understanding, developing and evaluating human-centred, effective, and quality care. As a minor pitfall, the case studies presented in the book are largely taken from the context of the UK, whereas a range of international cases might appeal to a more international audience. The authors, nonetheless, admit that knowledge of integrated care is situated in time and place and encourage feedback and interaction with their readers in the conclusion.

